# Exophytic Subserosal Uterine Adenomyomatous Polyp Mimicking Malignancy: A Case Report

**DOI:** 10.7759/cureus.43675

**Published:** 2023-08-17

**Authors:** Anu Manivannan, Monna Pandurangi, Radha Vembu, Sanjeeva Reddy

**Affiliations:** 1 Reproductive Medicine and Surgery, Sri Ramachandra Institute of Higher Education and Research, Chennai, IND

**Keywords:** endometriosis, infertility, dysmenorrhoea, extrinsic adenomyosis, adenomyomatous polyp

## Abstract

The epidemiological profile of adenomyosis has drastically changed in recent years due to advancements in imaging techniques. Even though adenomyosis is not uncommon in women of childbearing age, we present an intriguing case of a 30-year-old woman with long-standing progressive dysmenorrhea and infertility who had a posterior wall exophytic adenomyomatous polyp with full-thickness pseudo-invasion out of the uterine serosa into the right ovarian endometriotic cyst, mimicking malignancy. After surgical excision, the patient spontaneously conceived and delivered a live-term baby, soon after which she experienced an early recurrence. Clinicians must be aware of the distinctive features of different subtypes of adenomyosis to plan treatment and avoid invasive surgery.

## Introduction

Adenomyosis represents the benign invasion of the endometrial glands and stroma into the myometrium [1]. Because of advanced imaging techniques like transvaginal ultrasound (TVUS) and MRI, the diagnosis of adenomyosis has drastically evolved in recent years from being a histological entity into a complex clinical disease [2]. Adenomyosis affects approximately 20-30% [3] of women in reproductive years and 7.5-24.4% of infertile women undergoing assisted reproductive technology (ART) [4]. It is also frequently seen in women with advanced age, endometriosis, recurrent pregnancy loss, and recurrent implantation failure. We present an unusual case of recurrent exophytic adenomyoma, with pseudo-invasive polypoid growth, seen penetrating the serosa, mimicking malignancy.

## Case presentation

A 30-year-old Indian woman, nulligravida, married for one year, anxious to conceive, presented with long-standing, progressive, and severe dysmenorrhea, lasting up to five days of menses, not relieved with medical management. She had regular menstrual cycles with moderate flow. There was no history of chronic pelvic pain, dyspareunia, bowel, or bladder symptoms. Four years ago, she underwent a laparoscopic cystectomy for a symptomatic left ovarian endometriotic cyst measuring 11.1 x 7.8 x 8.8 cm.

**Figure 1:**
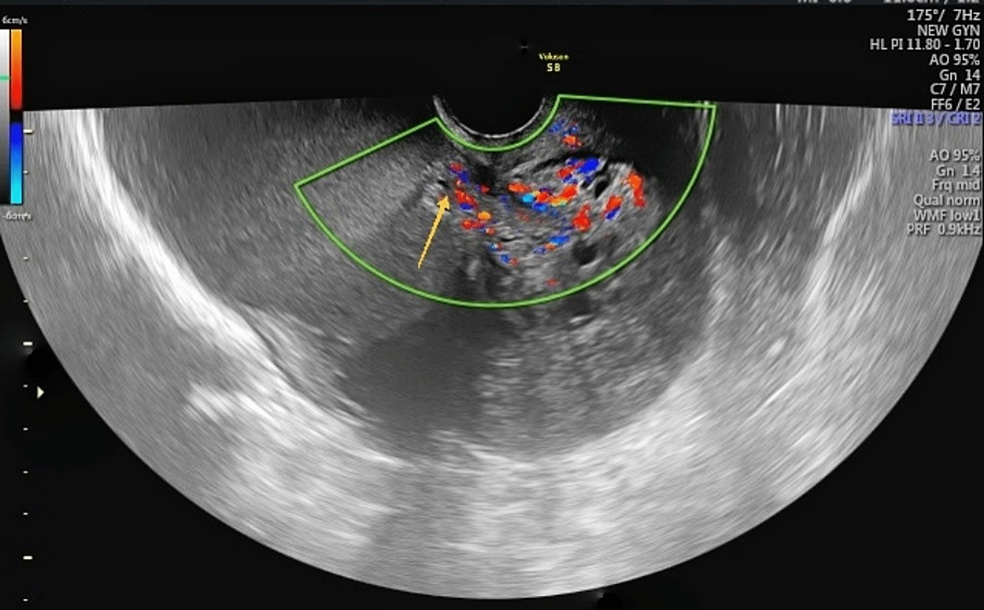
Ultrasound findings of exophytic adenomyoma TVUS image showing a focal ill-defined cystic heterogenous lesion (arrow) in the posterior uterine wall, penetrating the serosa with increased vascularity (Colour score: 3)

A bimanual pelvic examination was done, which suggested a retroverted uterus corresponding to 8-10 weeks of gravid uterus size, with posterior and right forniceal fullness, fixed to the uterus, and non-tender. No nodularity was felt in the pouch of Douglas (POD) and uterosacral ligaments. 2D and 3D TVUS was performed, which suggested a retroverted bulky uterus measuring 11.0 x 5.0 x 8.3 cm with a focal ill-defined cystic heterogenous intra-myometrial lesion measuring 5.2 x 4.1 cm in the posterior uterine wall, penetrating the serosa (Figure 1). An extra-serosal extension of 19 mm was noted, which appeared as a vascularized mass (Color score: 3) burying into the right ovarian endometriotic cyst of size 12.4 x 9.1 cm. A left ovarian endometriotic cyst measuring 3.0 x 2.5 cm was noted. Bilateral ovaries were stuck in the POD, and the sliding sign was negative. Minimal free fluid was present in the pelvis. The antral follicle count was two.

**Figure 2:**
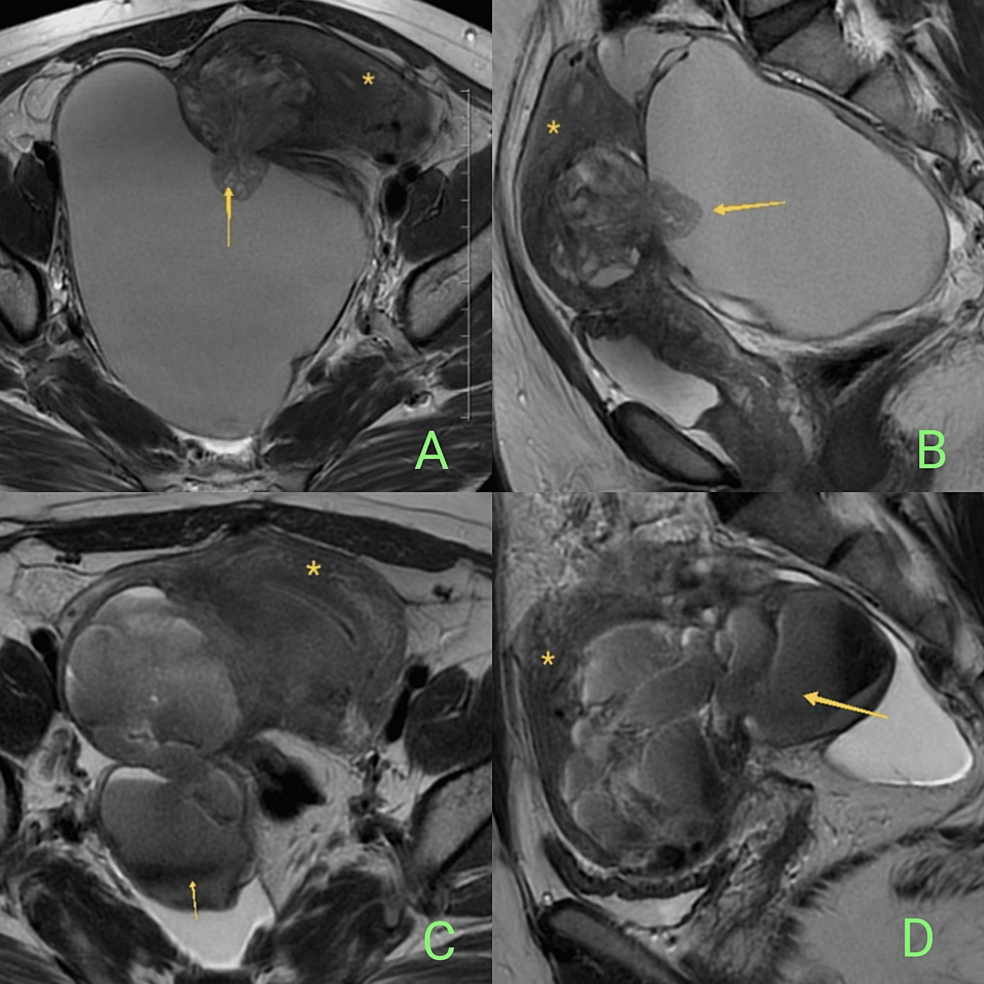
MRI findings of exophytic adenomyoma MRI T2-axial (A) and sagittal (B) images during the initial presentation depicting an exophytic adenomyomatous polyp (arrow) extending from the right posterolateral wall of the uterus (asterisk) abutting into the right endometriotic cyst. The JZ is intact. MRI T2-axial (C) and sagittal (D) images during the recurrence depict a cystic adenomyoma (arrow) at the same site with a posterior serosal breech communicating with the right endometriotic cyst.

MRI pelvis suggested a well-circumscribed heterogeneous mass in the right posterolateral uterine myometrial wall, measuring 3.8 x 5.1 x 5.1 cm, with multiple small cystic locules showing T2 shading and corresponding T1 hyperintensity within the mass (Figures 2A, 2B). It had an exophytic projection, breaching the uterine serosa and extending into the large right ovarian endometriotic cyst. The junctional zone (JZ) was intact. The rectum was compressed and displaced to the left side, suggesting rectosigmoid junction adhesions. No significant lymphadenopathy was noted. Minimal free fluid was present in the pelvis.

Serum cancer antigen (CA)-125 level was 249.50 U/mL, serum lactase dehydrogenase (LDH) was 176 U/L (reference range: 208-378 U/L), and anti-mullerian hormone (AMH) was 4.06 ng/mL. A presumptive diagnosis of extrinsic adenomyoma was made. The differential diagnosis was cystic degeneration of the fibroid, ovarian carcinoma, or uterine leiomyosarcoma.

**Figure 3:**
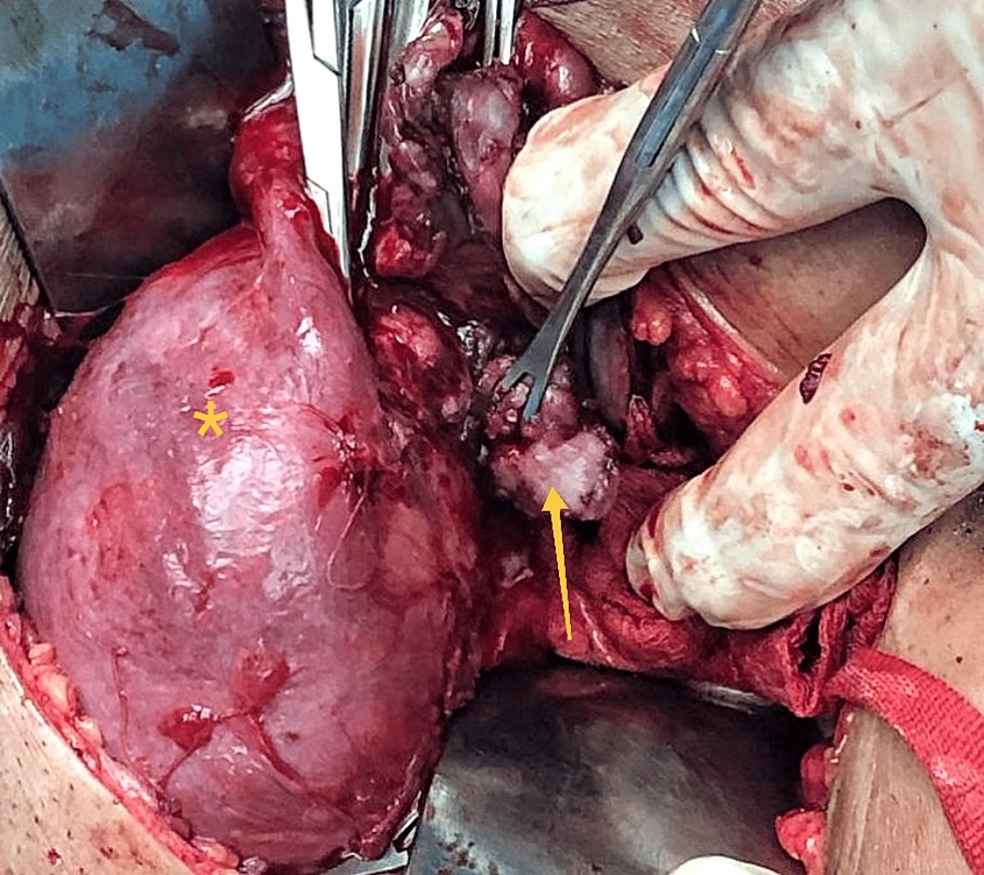
Exophytic adenomyoma Intraoperative finding of adenomyomatous polyp (arrow) breaching the uterine serosa (asterisk).

**Figure 4:**
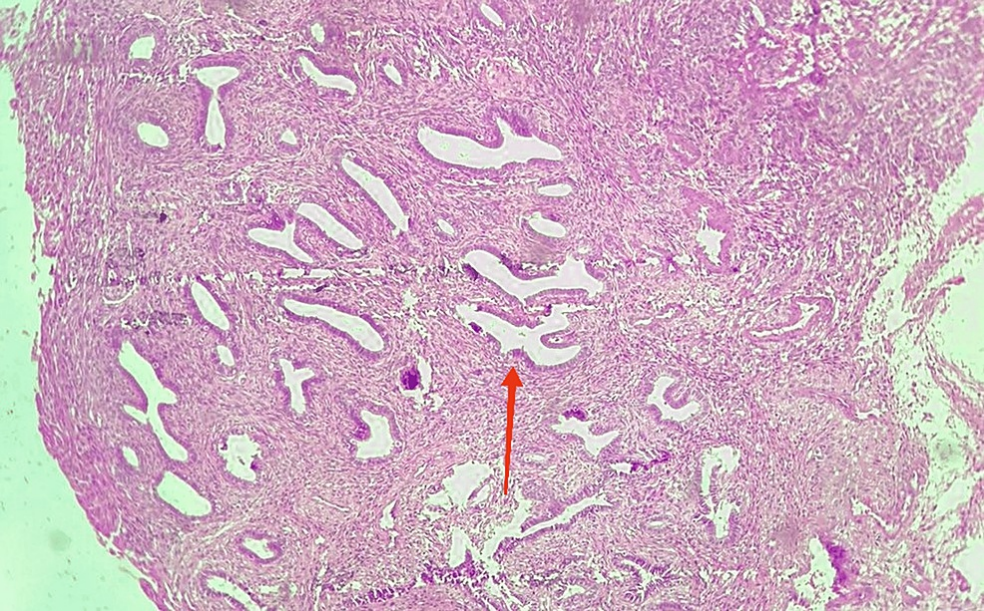
Histopathological image consistent with adenomyomatous polyp Histopathology of the excised tissue showing polypoidal tissue fragments with endometrial glands (arrow) surrounded by spindle-shaped smooth muscle cells and thick-walled blood vessels.

Given the concerning imaging features, the patient underwent laparotomy. Intraoperatively, the uterus was pushed anteriorly by a large right endometriotic cyst adherent to the posterior wall of the uterus and the rectosigmoid. After adhesiolysis and right endometriotic cystectomy, a polypoid soft lesion with intra-mural extension breaching through the right posterolateral uterine serosa was noted (Figure 3). The same was removed in toto, and the uterine defect was sutured. Bilateral tubes, along with fimbriae, were normal. The left ovarian endometriotic cystectomy was done. The excised tissue's histopathological examination was consistent with an adenomyomatous polyp (Figure 4). There was no evidence of atypia, mitosis, or necrosis. Thus, the patient had Stage IV (severe) endometriosis with an endometriosis fertility index (EFI) score of 7 out of 10, along with focal adenomyosis.

Post-surgery, the patient was advised periodic follow-up and assisted reproduction, given the increased risk of recurrence of adenomyoma and endometriosis, even though she had a higher EFI score. She was given one dose of leuprolide acetate 11.25 mg depot and advised to continue dienogest 2 mg tablets until she was ready to plan pregnancy.

The patient was lost to follow-up post-surgery. Two years later, she again reported to our clinic with complaints of severe dysmenorrhea. She stopped taking dienogest tablets and conceived spontaneously five months after the surgery. She delivered a live-term male baby weighing 2.8 kg through an elective lower-segment cesarean section six months ago. The antenatal, intrapartum, and postnatal periods were said to be uneventful.

TVUS followed by MRI pelvis was done, which was suggestive of a larger well-circumscribed multiloculated T2 hypointense mass at the same site, showing multiple fluid-fluid levels and areas of gradient recalled echo blooming, measuring 7.8 x 5.7 x 5.6 cm, suggestive of cystic adenomyoma, again with the posterior serosal breach, abutting the right endometriotic cyst, measuring 4.8 x 5.5 x 5.0 cm (Figure 2C, 2D). The lesion demonstrated T2 shading sign with the corresponding hyperintense signal on T1 weighted images. Sigmoid colon adhesions were noted along with bilateral endometriotic cysts.

**Figure 5:**
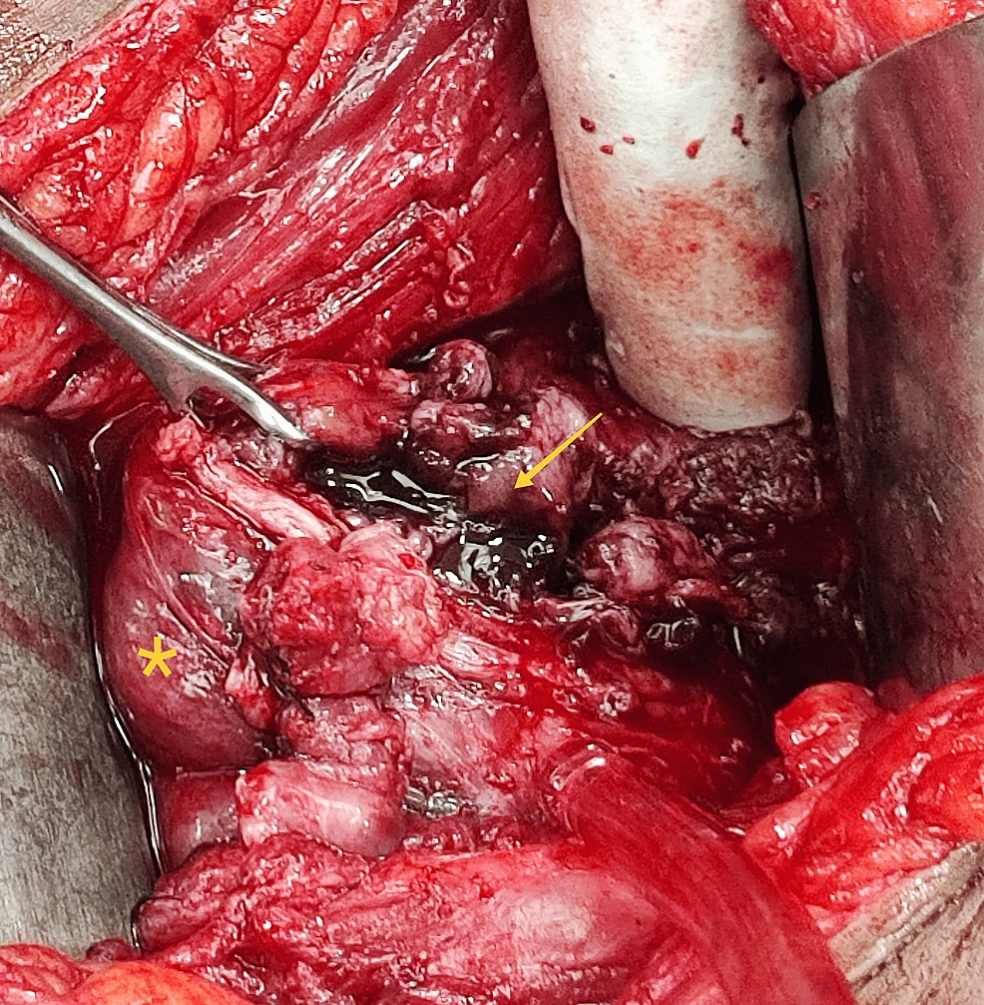
Surgical findings of recurrent adenomyoma Intraoperative findings show posterior uterine wall cystic adenomyoma (arrow) filled with chocolate brown colored fluid

The patient was advised total hysterectomy with bilateral endometriotic cystectomy because of the sizeable exophytic adenomyoma, recurrent endometriosis, and persistent severe pain. Since the patient insisted, we planned a fertility-preserving surgery. Extensive rectosigmoid adhesions were noted during laparotomy, and the same was released. Bilateral ovarian endometriotic cysts were excised. Chocolate-brown colored fluid drained from the exophytic cystic adenomyotic growth (Figure 5). It was excised in toto, and the posterior wall defect was sutured in two layers. Serosa was sutured using baseball sutures. There was no direct connection between the adenomyoma and the endometrial cavity.

Histopathology was once again consistent with adenomyoma. The patient was counseled for strict periodical follow-up and started on dienogest tablets (2 mg once daily) for six months. She was asymptomatic in the follow-up period, and we planned to continue long-term hormonal treatment until the patient plans for the subsequent pregnancy.

## Discussion

To the best of our knowledge, this is the first case of a pseudo-invasive posterior wall subserosal adenomyomatous polyp documented in the literature. Adenomyosis is a heterogenous condition that may manifest in various forms. There have been reports of adenomyomatous endometrial polyps in the literature, and it is essential to note that the risk of endometrial cancer is 16% with atypical endometrial polypoid adenomyoma [5]. In 1991, Sakamoto et al., in a study of 128 patients, suggested that subserosal adenomyosis may develop as a variant of pelvic endometriosis [6]. Few cases of subserosal adenomyoma [7-9] and about 46 cases of cystic adenomyosis [10] have been documented in the literature.

Kishi et al. [11] threw light on relatively new subtypes of adenomyosis as per MRI findings, namely subtype 1 (intrinsic), subtype II (extrinsic), subtype III (intramural), and subtype IV (indeterminate) adenomyosis, respectively. Intrinsic adenomyosis primarily affects the JZ, surrounded by healthy myometrium. Extrinsic adenomyosis develops in the outer third of the uterus, interrupting the serosa with JZ intact. It is usually identified as an ill-defined subserosal myometrial lesion with a hypointense signal on T2-weighted MRI, with high-intensity cystic areas in the center [12]. Intramural adenomyosis resides solitarily without disrupting the JZ and serosa. The indeterminate type consists of heterogenous mixtures of advanced cases of subtype I-III of adenomyosis and has no clear definition.

Extrinsic adenomyosis was found to be frequent in the posterior wall of the uterus (96.1%), closely associated with pelvic endometriosis with POD obliteration (96.1%) and ovarian endometrioma (66.7%). In 72.5% of extrinsic adenomyosis, a teardrop deformity of the rectum, in which the rectum is dragged up toward the uterus instead of the usual round shape, was observed during MRI. Marcellin et al. also reinforced that 56.5% of deep infiltrating endometriosis (DIE) patients had focal adenomyosis of the outer myometrium diagnosed by pre-operative MRI [13]. Their co-existence was associated with long-standing painful symptoms, infertility, multiple and severe DIE, and increased rates of surgical intervention for endometriosis and ovarian endometrioma.

Thus, it is hypothesized that pelvic endometriosis initially forms utero-rectal adhesion, resulting in the obliteration of POD, and then invades the rectum posteriorly and disrupts the uterine serosa anteriorly. This "outside-to-inside invasion" [14] from an ovarian endometriotic cyst into the posterior uterine wall resulted in an adenomatous polyp in this patient. It is in striking contrast to classical and most accepted theories about the pathogenesis of adenomyosis, namely the direct myometrial invasion of cells from the endometrium basalis due to hyperperistalsis, leading to tissue injury and repair mechanism and the de novo metaplasia of the embryonic pluripotent Mullerian remnants or adult stem cells into endometrial progenitor cells. Hence, this brings us to the novel concept of archimetriosis, which emphasizes uterine hypercontractility as a significant risk factor and progenitor contributing to both adenomyosis and endometriosis [15]. The prevalence of adenomyosis in women with endometriosis varies widely, ranging from 20% to 80% [4].

The presence of endometrioma poses a difficulty in counting the antral follicles by TVUS [16], as they can only be seen in the ovarian tissue located at the periphery of the cyst. We gave importance to the age and AMH in counseling this patient regarding fertility.

Fertility-preserving excision of focal adenomyosis can be done with either laparoscopy or laparotomy. Both of which have specific advantages and disadvantages. During surgery, the distinction between adenomyoma and healthy myometrium can be better done by palpation, facilitating complete excision. Given the concerning imaging findings, laparotomy was performed in this case. The aim was to thoroughly resect the lesions to prevent recurrence and meticulously reconstruct the uterine defect, which is more critical to avoid uterine rupture during subsequent pregnancy. Even though laparoscopy is associated with fewer postoperative complications, including pain, and shorter hospital stay, it also involves the risk of upstaging of malignancy if present, incomplete adenomyoma excision, inadequate reconstruction of the uterine wall, concerned use of the morcellator for retrieval of the specimen, and higher risk of uterine rupture during pregnancy when compared to laparotomy. The factors attributing to uterine rupture are the method of removing adenomyosis (e.g., cold knife, powered instruments), extent of resection, resultant myometrial defect and its proper reconstruction, postoperative complications, the interval between the surgery and pregnancy, and skill of the surgeon. The risk of uterine rupture during pregnancy following adenomyomectomy is more than 1.0% compared to 0.26% post-myomectomy [17]. Hence, shared decision-making, proper pre-operative consent, and proper information to the patient and physician during the pregnancy succeeding adeno-myomectomy are paramount.

For secondary prevention, we counseled the patient for regular follow-up and the need for long-term hormonal treatment. While choosing the medical treatment for endometriosis, a pragmatic approach must be followed considering the efficacy, side effects, tolerability, adherence to the treatment, costs, and women's preferences. Continuous oral low-dose monophasic estrogen-progestin hormonal contraception, progestogens like depot medroxyprogesterone acetate, medroxyprogesterone acetate, norethisterone acetate, desogestrel, and dienogest, or levonorgestrel-releasing intrauterine system can be recommended. The unfavorable side effect profile limited the long-term use of GnRH agonists and oral antagonists [18].

This patient had an exophytic adenomyomatous polyp, which progressed to a cystic adenomyoma despite the primary surgery and amenorrhea induced by pregnancy and lactation. Both are rare manifestations of adenomyosis. Hence, the distinctive imaging features and various MRI-based subtypes of adenomyosis [11,12], which can pose a diagnostic dilemma, must be recognized. Extrinsic adenomyosis was significantly correlated with early ovarian endometrioma recurrence in less than three years [19]. In co-existing endometriosis and adenomyosis cases, patients must be informed about its chronic nature, potentially affecting the long-term overall quality of life, including mental health, social activities, and partner relationships.

## Conclusions

The presence of an exophytic pseudo-invasive subserosal adenomyomatous polyp protruding into the ovarian endometrioma is a relatively rare condition. These lesions may mimic cystic degeneration of fibroid, leiomyosarcoma, and ovarian malignancy, borderline ovarian tumors. It highlights that pelvic endometriosis may be the precursor for extrinsic adenomyosis, and their co-existence represents a significant decisive point in integrating the diagnostic strategy and treatment planning. Clinicians must be aware of the distinct features of relatively new MRI-based subtypes of adenomyosis to avoid overdiagnosis and invasive treatment. Shared decision-making, proper pre-operative consent, adequate information to the patient and physician during the pregnancy succeeding adenomyomectomy, and long-term medical management are crucial.

## References

[1] Bird CC, McElin TW, Manalo-Estrella P (1972). The elusive adenomyosis of the uterus—revisited. Am J Obstet Gynecol.

[2] Chapron C, Vannuccini S, Santulli P (2020). Diagnosing adenomyosis: an integrated clinical and imaging approach. Hum Reprod Update.

[3] Bourdon M, Santulli P, Marcellin L (2021). Adenomyosis: an update regarding its diagnosis and clinical features. J Gynecol Obstet Hum Reprod.

[4] Vannuccini S, Petraglia F (2019). Recent advances in understanding and managing adenomyosis. F1000Res.

[5] Biasioli A, Londero AP, Orsaria M (2020). Atypical polypoid adenomyoma follow-up and management: systematic review of case reports and series and meta-analysis. Medicine (Baltimore).

[6] Sakamoto A (1991). Subserosal adenomyosis: a possible variant of pelvic endometriosis. Am J Obstet Gynecol.

[7] Raj T, DeWitt R, Smith NE (2018). Adenomyoma with pseudoinvasive growth pattern and serosal penetration mimicking endometrial carcinoma. Int J Gynecol Pathol.

[8] Takeda A, Imoto S, Sugiyama C, Nakamura H (2013). Uterine adenomyoma with exophytic subserosal growth: case report of rare manifestation with image diagnosis and laparoscopic-assisted excision. J Minim Invasive Gynecol.

[9] Ç EB (2008). Subserosal mass-like adenomyosis: Is it polypoid endometriosis. https://gcris.pau.edu.tr/handle/11499/41331.

[10] Xu T, Li Y, Jiang L, Liu Q, Liu K (2022). Subserous cystic adenomyosis: a case report and review of the literature. Front Surg.

[11] Kishi Y, Suginami H, Kuramori R, Yabuta M, Suginami R, Taniguchi F (2012). Four subtypes of adenomyosis assessed by magnetic resonance imaging and their specification. Am J Obstet Gynecol.

[12] Bazot M, Daraï E (2018). Role of transvaginal sonography and magnetic resonance imaging in the diagnosis of uterine adenomyosis. Fertil Steril.

[13] Marcellin L, Santulli P, Bourdon M (2020). Focal adenomyosis of the outer myometrium and deep infiltrating endometriosis severity. Fertil Steril.

[14] Chapron C, Tosti C, Marcellin L (2017). Relationship between the magnetic resonance imaging appearance of adenomyosis and endometriosis phenotypes. Hum Reprod.

[15] Leyendecker G, Wildt L, Laschke MW, Mall G (2023). Archimetrosis: the evolution of a disease and its extant presentation: pathogenesis and pathophysiology of archimetrosis (uterine adenomyosis and endometriosis). Arch Gynecol Obstet.

[16] Lima ML, Martins WP, Coelho Neto MA, Nastri CO, Ferriani RA, Navarro PA (2015). Assessment of ovarian reserve by antral follicle count in ovaries with endometrioma. Ultrasound Obstet Gynecol.

[17] Osada H (2018). Uterine adenomyosis and adenomyoma: the surgical approach. Fertil Steril.

[18] Barbara G, Buggio L, Facchin F, Vercellini P (2021). Medical treatment for endometriosis: tolerability, quality of life and adherence. Front Glob Womens Health.

[19] Sun M, Xu P, Zou G, Wang J, Zhu L, Zhang X (2021). Extrinsic adenomyosis is associated with postoperative recurrence of ovarian endometrioma. Front Med (Lausanne).

